# Enhancing safe routes to school programs through community-engaged citizen science: two pilot investigations in lower density areas of Santa Clara County, California, USA

**DOI:** 10.1186/s12889-019-6563-1

**Published:** 2019-03-01

**Authors:** Nicole M. Rodriguez, Alisa Arce, Alice Kawaguchi, Jenna Hua, Bonnie Broderick, Sandra J. Winter, Abby C. King

**Affiliations:** 1 Stanford Prevention Research Center, Department of Medicine, Stanford University School of Medicine, Stanford, CA 94305 USA; 2 Santa Clara County, CA Public Health Department, Center for Chronic Disease and Injury Prevention, San Jose, CA 95126 USA; 3 Division of Epidemiology, Department of Health Research and Policy, Stanford University School of Medicine, Stanford, CA 94305 USA; 4 Stanford Prevention Research Center, 1070 Arastradero Road, Suite 100, Palo Alto, CA 94304-1334 USA

**Keywords:** Active transport, School health, Children, Safe routes to school, Citizen science, Built environment, Walking, Bicycling, Transportation, Community engagement

## Abstract

**Background:**

While promoting active commuting to school can positively affect children’s daily physical activity levels, effectively engaging community members to maximize program impact remains challenging. We evaluated the initial utility of adding a technology-enabled citizen science engagement model, called *Our Voice,* to a standard Safe Routes to School (SRTS) program to enhance program engagement activities and student travel mode behavior.

**Methods:**

In Investigation 1, a prospective controlled comparison design was used to compare the initial year of the Santa Clara County Public Health Department’s SRTS program, with and without the *Our Voice* engagement model added, in two elementary schools in Gilroy, California, USA. School parents served as *Our Voice* citizen scientists in the SRTS + *Our Voice* school*.* In Investigation 2, the feasibility of the combined SRTS + *Our Voice* methods was evaluated in a middle school in the same district using students, rather than adults, as citizen scientists. Standard SRTS program engagement measures and student travel mode tallies were collected at the beginning and end of the school year for each school.

**Results:**

In the elementary school investigation (Investigation 1), the SRTS + *Our Voice* elementary school held twice as many first-year SRTS planning/encouragement events compared to the SRTS-Alone elementary school, and between-school changes in walking/biking to school rates favored the SRTS + *Our Voice* school (increases of 24.5% vs. 2.6%, *P* < .001). The Investigation 2 results supported the feasibility of using students to conduct SRTS + *Our Voice* in a middle school-age population.

**Conclusions:**

The findings from this first-generation study indicated that adding a technology-enabled citizen science process to a standard elementary school SRTS program was associated with higher levels of community engagement and walking/biking to school compared to SRTS alone. The approach was also found to be acceptable and feasible in a middle school setting.

## Background

During the past 30–40 years, daily physical activity levels among U.S. youth have gradually declined [1]. Walking to school, in particular, decreased from 41 to 13% between 1969 and 2001 [2], with only 35% of children in grades K-8 living within one mile of school reporting usually walking/biking to school [3]. This trend continues despite the fact that walking/biking to school contributes to healthy daily levels of physical activity [4]. Safe Routes to School (SRTS)--a national program promoting safe options for walking/biking to school [5]--has been shown to increase physical activity among school-aged children through supporting bicycle and pedestrian education, school wellness policies, and engineering improvements [6, 7]. U.S. government initiation of SRTS began with two funded pilot projects in 2000, which quickly led to the growth of grassroots SRTS activities throughout the U.S. [8]. The U.S. SRTS model is built on the “Five E’s” concept: education, encouragement, evaluation, enforcement, and engineering [5].

A key though often challenging aspect of SRTS program adoption and sustainability is initial and ongoing engagement among residents and community stakeholders. For example, promoting collaborative community-research partnerships has been reported to be a key program adoption strategy related to successful SRTS program adoption in Canada and the U.S. [9]. However, in an educational climate of increasing fiscal constraints, finding efficient methods for building such collaborative partnerships can be difficult. This is despite reports that investments in programs and infrastructure that facilitate walking and biking to school have the potential for reducing transport expenditures for school districts and families [10].

The first-generation investigations described in this article evaluated an evidence-based community citizen science approach, called *Our Voice* [11]*,* for increasing community member and stakeholder engagement and retention in a local public health department-sponsored SRTS program which was being initiated in Gilroy, California, USA. The *Our Voice* approach combines the active community engagement of community-based participatory research with the standardized resident-based data collection methods that are a hallmark of citizen science [11]. The term “citizen science” refers to actively engaging local residents in gathering, analyzing, and utilizing data to improve community health and wellbeing [11]. In the *Our Voice* approach, residents learn to use the Discovery Tool (DT), a mobile environmental assessment app, to capture their walking route and take geo-coded photos and record audio-narratives of barriers and facilitators to health and wellbeing in their communities [12]. The DT (described below) is the first step in a multi-phase *Our Voice* citizen science process that includes data-driven community discussion and topic prioritization, and resident activation through advocacy with local stakeholders [11, 13–18]. While *Our Voice* has been employed successfully in a growing number of U.S. and global sites to improve local environments for physical activity and other health-enhancing behaviors [11, 15, 19], this is the first systematic application of *Our Voice* to school settings.

In Investigation 1 of this study, two elementary schools (grades kindergarten through grade 5) were compared, one with *Our Voice* added to the Santa Clara County, California Public Health Department’s (SCCPHD) standard SRTS program and one with the SRTS program alone. The major study question concerned whether SRTS + *Our Voice* was associated with increased school-wide SRTS engagement activities and greater changes in the number of students walking/biking to school relative to SRTS-Alone. SRTS program engagement activities continued to be tracked during the subsequent year to assess how well the activities begun in the initial SRTS year were maintained.

Additionally, since school districts in the U.S. often include both elementary schools (kindergarten through grades 5 or 6) and intermediate or middle schools (grades 6 or 7 through grade 8) [20], we explored the initial acceptability of including the citizen science engagement model in a SRTS program being initiated in a middle school in the same district (Investigation 2). The objective of Investigation 2 was to assess the initial feasibility and acceptability of the combined SRTS + *Our Voice* methods in a middle school setting when using middle school students themselves (rather than parents) as the citizen scientists.

## Methods

### Study location

Gilroy, California (total population = 53,231 in 2015) [21]—a lower density region of Santa Clara County, California—consists largely of farmland and suburban areas. High school graduates or higher represent 77% of the population, while 15% of the population lives in poverty [21]. Fifty-eight percent of residents self-identify as Hispanic/Latino, 32% identify as non-Hispanic White, and 26% are foreign-born. There are 18 public schools in the Gilroy Unified School District, which enrolls about 11,225 students [22].

In 2015, parental attitude and behavior survey data were gathered on 4117 parents across 12 Gilroy district schools by the Santa Clara County Public Health Department (SCCPHD) Safe Routes to School program, using forms from the National Center for Safe Routes to School (http://archive.saferoutesinfo.org/data-central). While, overall, many families living in the school district (54%) reported living within walking or biking distance from school, most families (69%) drove their children to school. Walking (19%), school buses (5%), carpools (4%), and biking (2%) were low. Reasons parents provided for not allowing their children to walk/bike to school included concerns about safety of intersections (54.8%), distance (54.5%), traffic speed (51.5%), violence/crime (51.4%), and traffic amount (48.7%) [23].

In 2015, investigators from Stanford University partnered with the Santa Clara County Public Health Department (SCCPHD) to conduct an initial evaluation of the *Our Voice* citizen science approach as an addition to SCCPHD’s SRTS program.

## Investigation 1: Comparison of elementary schools with and without Our Voice

### Design and school site selection

A prospective controlled comparison design was used. During September 2015, SCCPHD staff identified, with assistance from the Gilroy Unified School District, the next two Gilroy elementary schools that were ready to initiate SRTS for the first time. Given that the two schools were on somewhat different time tables for initiating SRTS activities, School A (which was ready to initiate SCCPHD’s SRTS activities in late September 2015) was chosen to receive the SRTS + *Our Voice* program, while School B (which was ready to initiate SRTS activities in November 2015) received SRTS-Alone.

At Elementary School A (SRTS + *Our Voice)*, six local adult “citizen scientists” were recruited through school parent-teacher groups and school staff meetings. At Elementary School B, standard SRTS data collection was conducted by SCCPHD staff with assistance from five parent and community volunteers. Human subject approvals were obtained from the Institutional Review Boards (IRBs) at Stanford University and SCCPHD.

### SRTS program intervention activities

The SRTS intervention program typically begins with a standard set of observational evaluations of the local environment. SRTS program walkability/bikeability observations [24], conducted in Fall 2015 by SCCPHD staff and several community members, included observational surveys of student pedestrian and bicycle helmet and safe riding behaviors, and environmental observation audits (i.e., evaluating features facilitating or hindering safe walking/biking). At both sites, the pedestrian/bicyclist observational surveys were conducted as part of standard SRTS practice in this locale [24]. In contrast, collection of the SRTS environmental observation audit data differed between the two schools. At the SRTS + *Our Voice* site, the SRTS environmental observation audit tool was replaced with the DT mobile app. It was hypothesized that this app would allow community members to document relevant environmental barriers and facilitators in more depth by using photographs and personal narratives to contextualize resident observations.

Six parents at School A were trained to conduct DT assessments (lasting 30–45 min each) on two mornings prior to school start during one week in September 2015. DT walking zones were selected by a team of SCCPHD staff and community members and focused on areas most frequently used by students traveling to school. Participants were given a prompt to capture aspects of their environment that made it easier or harder to walk/bike to school and were asked to take photos and accompanying audio narratives in their designated zones. DT training required less than 10 min and was accomplished just prior to its use.

At School B, the typical SRTS environmental audit tool was used instead of the DT [24]. This audit tool includes 50 questions measuring the following eight factors on a scale of 1 to 7, with 7 classified as a serious problem: room to walk, ease of street crossing, room to bicycle, ease of bicycling, parent support, driver behaviors, safety, and pleasantness/aesthetics. Auditors check off and rate each item as they walk in their designated zones. Five adult community members (three parents and two after-school staff) participated in the assessments along with SCCPHD staff. Assessments lasted approximately 30–45 min and were conducted in the morning prior to school start on two days within the same week as School A. As with the selected DT zones, the environmental assessment zones for School B were selected by a team consisting of SCCPHD staff along with members of the school community and focused on areas most frequently used by students coming to school.

#### Community meetings to discuss the environmental audit information

In School A, following DT use, standard *Our Voice* methods were deployed, including two community meetings where citizen scientists categorized data, prioritized issues, and advocated with stakeholders for realistic changes related to those identified issues [11]. The community meetings, facilitated by SCCPHD, were held 1–2 months after the DT walks. At the first meeting, each participant received a data packet with printed photographs and transcripts from their DT walks that had been transcribed by the research team. Citizen scientists thematically categorized their data within a theme, for example, traffic, crosswalks, stop signs, safety, and pathways. After thematically categorizing their data, participants ranked the themes based on importance and feasibility. Relevant local stakeholders were also identified for subsequent engagement (e.g., school administrators, public works engineers).

At the second meeting with community stakeholders, the citizen scientists, with support from SCCPHD staff, visually presented the *Our Voice* methods and results to their stakeholders and brainstormed ideas for realistic local improvements. In subsequent meetings initiated by the citizen scientists across the school year, they continued to explore ways of improving the school environment in addition to instituting action plans to address key concerns.

In School B, per standard practice in the County, SCCPHD staff reviewed and analyzed data from the SRTS observational assessment tools and developed a slide show with photos that displayed data from the environmental assessments at this school’s stakeholder meeting. Consistent with SRTS program methods in this locale, information was presented by the SCCPHD staff and included brainstorming of ideas and prioritization with key stakeholders. As per the standard SRTS protocol, this constituted the only meeting in this school.

### Project assessments

#### SRTS program engagement assessment

In order to measure program engagement at both elementary schools during this initial SRTS year, the frequency of meetings, events, and activities were documented over the 9-month school period from September 2015 to May 2016 by SCCPHD contractors and SRTS Parent Coordinators who reported to school staff and the SCCPHD. Tracked events included SRTS meetings and planning activities, educational activities, and “encouragement” events (see Table 2).

#### Student travel mode assessment

At the beginning and end of the school year, SCCPHD staff sent a packet of standard SRTS Student Travel Tally forms to each elementary school [24, 25]. For both schools, teachers were instructed to collect the Travel Tally forms at least twice during the Tuesday to Thursday period of the week of September 15–17 (beginning of the school year) and during the Tuesday to Thursday period of the week of May 3–5 (end of the school year), with the tally data averaged within each time period for each school. Because the travel tally weeks occurred at the same time for both schools, extraneous factors that could have potentially affected walking/biking to school (e.g., weather) were held constant. Teachers were responsible for surveying their students on whether they walked, biked, or rode in a vehicle to school on the above specified days during each travel tally week. SCCPHD staff retrieved the surveys and entered the information onto the SRTS National Data Center website, which is a secure online central database system.

#### Exploring maintenance of program engagement activities during the following year

As is common practice for SRTS, schools are encouraged to continue to work on SRTS action plans and activities developed during the initial program year. SCCPHD staff continued to provide technical assistance and resources as needed, and track numbers and types of SRTS program activities that occurred.

### Data analysis

Between-school differences in the percentage of children walking/biking to school at the beginning and end of the year were evaluated using Pearson’s chi-square test, with alpha set at .05, two-tailed. To capture the differences in baseline proportions and enhance the robustness of the data analysis for the student travel tally data, a “difference in differences” (DID) analytic procedure [26] also was conducted to evaluate the additional treatment effect of the *Our Voice* intervention on the proportions of student who walked/biked to school. DID was conducted by using the following logistic model with a simulated dataset where the total numbers of students and travel tally data were applied as parameters.

$$ logit\left( wb=1\right)={\beta}_0+{\beta}_tt+{\beta}_e ov+{\beta}_{\ast }t\ast ov $$

where *wb* is walk/bike, *t* is for time (baseline/pre vs. endline/post), and *ov* is for *Our Voice* intervention (SRTS + *Our Voice* vs. SRTS only). DID is *β*_∗_, the interaction term coefficient. *β*_∗_ is then exponentiated to obtain the odds ratio or effect size. All statistical analyses were performed using STATA 15.0 Statistical Software [27].

The total number of SRTS program engagement activities occurring across the year in each school is presented in a descriptive format (Tables 2 and 4).

## Results

### School descriptions

The two elementary schools, which included grades kindergarten to fifth grade, had similar characteristics with respect to general student population size (total student population = 740 in School A and 730 in School B) and student gender distribution (~ 50% each of males and females). Based on the 2015 parental survey, the two schools were also generally similar with respect to the majority of students being driven to and from school in a family vehicle (82% in School A and 71% in School B), the proportion of parents reporting that their child’s school encouraged walking or biking to and from school (36% in School A and 33% in School B), and parental perceptions that walking/biking to school was healthy for their child (83% in School A and 89% in School B). With respect to general socioeconomic characteristics, California Department of Education enrollment statistics indicated that during the 2015–16 school year, 16.5% of children in School A were non-Hispanic white, 67.8% were of Latino or Hispanic descent, and 5.8% were of Asian descent. Based on household income, 48.2% of children in School A were eligible for the National Free or Reduced-price School Lunch Program based on household income. Meanwhile, In School B, 47.1% were non-Hispanic white, 39.4% were of Latino or Hispanic descent, and 7.9% were of Asian descent. Based on household income, 22.4% of children in School B were eligible for the National Free or Reduced-price School Lunch Program based on household income.

### Pre-SRTS program SCCPHD school transportation data

September, 2015 pre-program travel mode data collection conducted on the total School A student population (*N* = 740) revealed that 94.7% (*n* = 701) of all students traveled to school by car, 5.0% (*n* = 37) walked to school, and 0.3% (*n* = 2) traveled to school by bicycle. Of the total School B student population (*N* = 730), 82.3% (*n* = 601) of all students traveled to school by car, 17.0% (*n* = 124) walked to school, and 0.7% (*n* = 5) arrived at school by bicycle. These percentages indicate that pre-intervention walking or biking to School A was significantly lower at the beginning of the school year relative to walking or biking to School B, with a between-school chi-square comparison for the above percentages of *X*^*2*^ = 8.3 [df = 1], *P* = .004.

### Citizen science participant demographics

Citizen science participant demographic information was collected in post-assessment surveys from School A parent participants. Participants had an age range of 46–49 years, and five of the six were women. When asked about race/ethnicity, four identified as Hispanic White and two as non-Hispanic White or Other. Demographic information was not available for adults participating in School B SRTS assessments because the standard SRTS process did not involve such surveys.

### Identification of school environment challenges through the observational audits

From the *Our Voice* citizen scientist discussions based on the DT photographs and audio narratives collected at School A, the top three environmental barriers to walking/biking to school identified by the citizen scientists were concerns with traffic flow, sidewalk issues, and a specific problematic intersection. In School B, the SRTS environmental audit tool indicated somewhat higher (worse) scores on driver behaviors, ease of street crossings, ease of bicycling, and parent support (all received mean scores of 3 on the 1- to 7-point scale).

### Stakeholder engagement in SRTS at each school

The School A SRTS + *Our Voice* stakeholder meeting engaged 13 stakeholders from seven different community sectors (see Table 1). School B’s standard SRTS advocacy meeting engaged 12 stakeholders from six different sectors (Table 1).

**Table 1:** Stakeholder sectors represented at community stakeholder advocacy meetings at the two elementary schools

Stakeholder Sectors	School A (SRTS + *Our Voice)*	School B (SRTS Alone)
Teachers	0	0
School Administration	1	1
Students	0	0
Parents	3	5
Public Works	1	0
Public Health	3	2
Law Enforcement	1	1
Bike Advocate	2	1
School District	0	0
Research team	2	0
Community Members	0	2

### SRTS program engagement activities

As summarized in Table 2, School A reported twice as many SRTS team meetings and action planning activities (*n* = 8) compared to School B (*n* = 4). These included the initiation of monthly SRTS task force meetings at School A and a 3-year Safe Routes to School Action Plan generated by parents. A team of six School A parents became involved in managing the SRTS program, while at School B, parent volunteers could not be identified, so a teacher took on the responsibility of coordinating SRTS activities. School A also reported more SRTS events than School B **(**Table 2**)**, and as a result of increased interest in biking to school, installed two additional bike racks. No such environmental changes were observed at School B.

**Table 2:** Community engagement at the two elementary schools across the initial 9-month school period (Fall 2015 to Spring 2016)

Engagement metrics	Definitions	School A (SRTS + *Our Voice)*	School B (SRTS Alone)
Meetings and Action Planning (counts)			
Community meeting	Meeting to identify and prioritize key issues	1	0
Stakeholder meeting	Meeting to present findings to local stakeholders	1	1
SRTS team meeting	Follow-up meetings involving citizen scientists and community residents interested in SRTS	5	2
Action Plan	Action plan developed by community residents to further local SRTS	1	1
Total meetings		8	4
Encouragement Events (counts)			
Walk/bike events	School-wide events such as National Bike to School Day and Bike Education Days	2	1
Walk & Roll Days	Days set aside to encourage walking and biking to school	5	2
Walking school bus	A walking school bus involves a group of students and parents walking together on the way to school in the morning	5	2
Total encouragement events		12	5
Educational Activities (counts)			
In-class educ. Activity: Grades K, 2nd, 4th, or 5th	Educational activities supported by SRTS in a classroom setting	12	13
Bike Rodeo	Event that includes biking lessons and safety tips	0	1
Family Fun Bike Night / Bike Repair	Event supported by SRTS to teach students about biking and bicycle repair	1	0
Traffic safety educ. & helmet distribution	Event supported by SRTS to teach students about traffic safety and distribute helmets	1	0
Total educational activities		14	14
OVERALL NUMBER OF SRTS ACTIVITIES		34	23

The frequency of overall activities noted above favored the SRTS + *Our Voice* school, which had a 48% greater number of SRTS program engagement activities than the SRTS-Alone school (see Table 2).

### Changes in SRTS student travel mode data across year 1

As summarized in Table 3, at School A, the SRTS tally forms capturing the entire student population indicated that walking/biking to school increased from 5.3% (5.0% walked, 0.3% biked) to 30% (22.0% walked, 8.0% biked), while walking/biking rates at School B, collected in the same manner, decreased slightly from 17.7% (10.8% walked, 0.7% biked) to 15.0% (13.0% walked, 2.0% biked). Pearson’s chi-square test indicated that the between-school difference was statistically significant (*X*^*2*^ = 12.3 [df = 1], *P* < .001), with endpoint walk/bike rates twice as high in School A relative to B. Furthermore, “difference in differences” (DID) modeling indicated an effect size of 9.34 (*P <* 0.001; 95% confidence interval = 5.92–14.67), which indicates that the SRTS + *Our Voice* program, compared to the SRTS-Alone program, increased the odds of students walking/biking to school by a factor of 9.34.

**Table 3:** One-year changes in SRTS student travel mode data

	School A (SRTS + Our Voice)			School B (SRTS only)			
	pre	post	post-pre difference	pre	post	post-pre difference	diff in diffs (DID)
Traveled by car - %	94.7%	70.0%	−24.7%	82.3%	85.0%	2.7%	−27.4%
Walked - %	5.0%	22.0%	17.0%	17.0%	13.0%	−4.0%	21.0%
Bicycled - %	0.3%	8.0%	7.7%	0.7%	2.0%	1.3%	6.4%
Walked/bicycled - %	5.3%	30.0%	24.7%	17.7%	15.0%	−2.7%	27.4%

### Maintenance of SRTS program engagement activities following the initial SRTS year

Table 4 summarizes the SRTS program engagement activities occurring at each school during the following school year (Fall, 2016 to Spring, 2017). As shown in this table, School A had an almost three-fold greater number of SRTS-related meetings and activities relative to School B.

**Table 4:** Community engagement at the two elementary schools across the subsequent 9-month school period (Fall 2016 to Spring 2017)

Engagement metrics	Definitions	School A (SRTS + *Our Voice)*	School B (SRTS Alone)
Meetings and Action Planning (counts)			
Community meeting	Meeting to identify and prioritize key issues	0	0
Stakeholder meeting	Meeting to present findings to local stakeholders	1	0
SRTS team meeting	Follow-up meetings involving citizen scientists and community residents interested in SRTS	7	2
Action Plan	Action plan developed by community residents to further SRTS efforts in their local school	1	1
Total meetings		9	3
Encouragement Events (counts)			
Walk/bike events	School-wide events such as National Bike to School Day and Bike Education Days	2	1
Walk & Roll Days	Days set aside to encourage walking and biking to school	16	2
Walking school bus	A walking school bus involves a group of students and parents walking together on the way to school in the morning	16	1
Total encouragement events		34	4
Educational Activities (counts)			
In-class educ. Activity: Grades K, 2nd, 4th, 5th	Educational activities supported by SRTS in a classroom setting	13	13
Bike Rodeo	Event that includes biking lessons and safety tips	0	0
Family Fun Bike Night / Bike Repair	Event supported by SRTS to teach students about biking and bicycle repair	1	0
Traffic safety educ. & helmet distribution	Event supported by SRTS to teach students about traffic safety and distribute helmets	1	0
Total educational activities		15	13
OVERALL NUMBER OF SRTS ACTIVITIES		58	20

## Investigation 2: Exploring the feasibility of the SRTS + *Our Voice* program in a middle school environment

## Methods

To further explore the feasibility of adding *Our Voice* to SRTS activities, the SRTS + *Our Voice* intervention was initiated at a middle school in Gilroy (student population size = 855) which had not participated previously in SCCPHD SRTS programming and for which SCCPHD was planning an SRTS intervention during a time period similar to that of the elementary schools (Fall, 2015 to Spring 2016). The middle school students themselves, rather than parents, served as citizen scientists. Participants from the middle school (grades 6 to 8), consisted of students and school faculty, recruited through a student leadership group, who were interested in engaging with SRTS. Human subject approvals were obtained from the Institutional Review Board (IRB) at Stanford University and from the IRB at the SCCPHD.

The SCCPHD SRTS program was generally similar to the program conducted at the elementary schools, with the exception that no in-class SRTS educational activities were delivered. Specific walking locations of particular relevance to school access were selected by SCCPHD staff in which to conduct the *Our Voice* DT assessments. The citizen scientists were 26 middle school students.

The middle school intervention and assessment methods were similar to those conducted at elementary school A and included the use of the DT mobile app (by the middle school students) to capture relevant barriers to and facilitators of active transport to school. The community meetings followed the same structure as those at elementary school A, (i.e., group-based DT photo categorization, discussion, and consensus-building around prevalent barriers to walking/biking to school; a subsequent student citizen scientist presentation of their data and insights to relevant local stakeholders with whom they brainstormed potential solutions and next steps).

SRTS program engagement measurement and SRTS Student Travel Mode data collection followed the same procedures as described earlier for the elementary school evaluation.

### Results

#### School description

California Department of Education enrollment statistics for 2015–2016 reported that 25.3% of middle school students were non-Hispanic white, 58.4% were of Latino or Hispanic descent, and 4.9% were of Asian descent. Based on household income, 47.6% were eligible for the National Free or Reduced-price School Lunch Program.

#### Pre-SRTS program school transportation data

Parental attitude and behavior survey data gathered in 2015 by the SCCPHD SRTS Program revealed that of the 855 parents surveyed, 86.2% (*n* = 737) reported that their children traveled to school by car, 10.4% (*n* = 89) walked to school, and 3.4% (*n* = 29) biked to school.

#### *Our Voice* participant demographics

Seventeen of the 26 students participating in *Our Voice* middle school activities completed demographic surveys. Reasons for survey non-completion included limited time allowed to complete the surveys in class and students failing to return the survey.

Participants had an age range of 12–13 years and consisted of 12 girls and five boys. When asked about race/ethnicity, six self-identified as non-Hispanic white.

#### Identification of school environment challenges through the observational audits

The top three barriers to walking/biking to school identified by citizen scientists via the DT and discussed during the first *Our Voice* meeting were traffic violations, safety concerns, and lack of crosswalks. Additional barriers included lack of pedestrian/bicyclist education, traffic congestion, lack of appropriate and visible traffic signs, trash, broken sidewalks, and lack of bike racks.

#### Stakeholder engagement in SRTS + *Our Voice* activities

The stakeholder advocacy meetings engaged the middle school students as co-presenters who described their findings to relevant stakeholders. A total of 16 stakeholders from nine different sectors participated, including from the school administration, Gilroy City Public Works and Police Departments, and non-governmental organizations (i.e., the Silicon Valley Bicycle Coalition).

#### SRTS program engagement activities

A total of 14 engagement activities were recorded across the school year, including four SRTS team meetings. SRTS educational activities included a school-wide bike-to-school education/encouragement day (40 students), and a traffic safety education and helmet distribution activity (75 students). The middle school also incorporated peer-to-peer student education (e.g., peer leaders demonstrated helmet fitting).

The SRTS + *Our Voice* intervention created opportunities to participate in new youth advocacy and outreach activities. For example, students were invited to speak at a Youth for Environment and Sustainability Conference in Berkeley, California and present their findings to the City of Gilroy Bike and Pedestrian Commission. Students continued to discuss SRTS topics throughout the school year as part of their monthly leadership group meetings.

Students also discussed SRTS-relevant environmental and infrastructure changes with key stakeholders during the advocacy meetings. Possible changes included a City Public Works Department evaluation of the flow of bikes, pedestrians, and cars entering and leaving the school area to increase safety. However, with the departure of the City’s Public Works engineer, such infrastructure changes were unable to be pursued at that time. Subsequently, SCCPHD staff reached out to the local Bicycle and Pedestrian Commission to build collaborations for such SRTS activities with youth advocacy support.

#### Changes in SRTS student travel mode data across the school year

Pre-post SCCPHD-collected travel mode tally data indicated that walking/biking to school rates from September 2015 to the end of the school year in 2016 remained at 19% at both time points (15.5% walked, 3.5% biked). A year after the SRTS assessments, it was observed that a new cohort of students had continued the discussion of SRTS topics and was planning activities to further promote walking and biking to school.

## Discussion

A commonly reported issue in SRTS programming is difficulty attracting community engagement and ongoing participation. Adding the *Our Voice* citizen science approach to the initial implementation of a SRTS elementary school program was associated with a significant increase in reported walking/biking to school relative to a comparison school which did not receive this additional intervention. The SRTS + *Our Voice* intervention was also generally associated with higher levels of engagement among elementary school parents across a 21-month period. Anecdotal observations by the SCCPHD staff members facilitating the SRTS programs suggested that the potential mechanisms that may have led to this increased engagement included an enhanced sense of ownership over local data, an increased interest in SRTS facilitated by directly capturing local barriers and facilitators with an innovative mobile app, and the community members’ role as co-presenters at stakeholder meetings. While it was beyond the scope of the current pilot investigation to capture such mechanisms in a more systematic way, *Our Voice* studies conducted in other community settings (e.g., neighborhoods) suggest that the civic engagement engendered by this type of citizen science approach may increase such mechanisms [11, 17, 18].

The size of the relative increase in reported walking/biking to school in the elementary school receiving SRTS + *Our Voice* (from 5.5 to 30%) was larger than what is typically reported in the SRTS literature (i.e., from 12.9 to 17.6%) [7]. It is possible that some source of measurement error affected these data, although standard SRTS tally procedures were used [25], and all students in each elementary school were included in these tallies. Given the preliminary nature of this investigation, it is important that the SRTS + *Our Voice* intervention procedures be replicated in other schools to better ascertain where the true impact of the additional *Our Voice* citizen science component may lie.

Results from applying the SRTS + *Our Voice* approach in a middle school suggest that youth as well as adults can fully participate in this type of citizen science process. Incorporating students in the assessment process proved successful in achieving high initial engagement (26 participating students), compared to citizen science engagement in the elementary school (six participating parents). SCCPHD staff also reported that engaging youth in the stakeholder presentations yielded more compelling stories, with the students themselves advocating for the improvement of their school environment. However, the middle school setting also presented structural challenges in carrying out some aspects of SRTS (e.g., teachers had limited time to supervise group meetings). While the students were invited to present at a youth environmental sustainability conference, they were unable to attend due to liability concerns. Despite these institutional barriers, the middle school students have continued to engage with the SRTS program. The initial cohort of middle school citizen scientists, who have moved on to high school, passed on responsibilities to a new cohort of middle school students who continue to lead school-based SRTS activities.

A large-scale study of 5-year SRTS program effects across 4 U.S. regions indicated that engineering and related environmental changes were associated with an 18% relative increase in walking and biking to school [6]. While the SRTS + *Our Voice* elementary school program resulted in at least one environmental change (i.e., additional bike racks), most activities involved new educational and motivational programs. The same large-scale study reported that such educational and encouragement programs have led to a 25% increase in walking and biking over a 5-year period [6]. In contrast, the middle school included specific targeting of local built environments and policies aimed at safer walking/biking to school. These included proposed solutions to improve the school’s traffic and drop-off policies. Unfortunately, with the departure of a key stakeholder in this area (the City’s Public Works engineer), such solutions were unable to be pursued during the project period. While environmental and policy changes may take longer to implement, they could potentially translate into more enduring changes in SRTS behaviors over time [28].

### Limitations

While the results of this first-generation investigation are promising, further investigation and replication of the effects of this combined approach in a larger and more diverse number of schools are needed. Among the limitations of the study design is the fact that the SRTS county health department initiated the SRTS program in School A approximately 6 weeks earlier than School B. This somewhat longer time period could have conceivably influenced the differential results favoring School A, which received the additional *Our Voice* intervention. During this 6-week start period, School A SRTS activities consisted of one Santa Clara County Public Health Department-led SRTS media day occurring on October 7, 2015. The Public Health Department staff promoted walking and biking to the school on that day. While it is not possible, given the study design, to determine the specific impacts of this one-day event on the differential walk/bike tallies observed at the end of the school year, there is little evidence in the literature indicating that a one-day SRTS event at the beginning of the school year could have such a substantial effect nine months later [7]. In addition, we observed even stronger between-school differences in SRTS engagement activities during the follow-up year when there was no difference in the SRTS program start date.

It is also possible that the larger proportions of lower income and racial/ethnic minorities in School A could have in some way resulted in the significant differences in walk/bike patterns shown across the school year in School A vs. B independent of the *Our* Voice intervention. However, prior SRTS research on U.S. schools that have larger proportions of Hispanic as well as lower-income students typically have found generally higher rates of walking/biking to school in those populations [7, 29], which was not the case at baseline in School A. This might have been due, at least in part, to the less urbanized nature of the school district under study relative to a number of other investigations, as well as the observation that lower-income Hispanic children living in western areas of the U.S. are more likely to live in less safe neighborhoods with poorer street environments [30]. These potential factors deserve further systematic investigation [7]. Similarly, while there could have been initial differences in parental and school-based readiness between the two elementary schools, pre-intervention parental survey data related to parental perceptions that their child’s school encouraged walking/biking to and from school and that walking/biking to school was healthy for their child were similar. In addition, the significant increase in walking/biking to school in the SRTS + *Our Voice* school, which had significantly lower baseline walking/biking rates relative to the SRTS-Alone school, was substantially larger than what one might typically expect if simply a “regression to the mean” phenomenon was operating.

Systematically tracking behavior change of this type across more extended time periods also presents challenges. The SRTS walking/biking travel mode tally required teachers to facilitate data collection, which can lead to teacher burden. Given the inevitable variability in school populations, policies, and resources that occur over time, SRTS programs may need to track walking/biking rates over much longer time spans (e.g., 5–10 years) using more rigorous experimental designs to accurately determine significant behavior changes [5]. An important goal of such programs is to ensure that the community does not disengage when change does not happen immediately.

## Conclusions

This first-generation study suggests that the addition of a technology-enabled citizen science process to a standard elementary school SRTS program was associated with higher levels of community engagement and reported walking/biking to school compared to SRTS alone. The results also indicate that this approach is feasible to employ in a middle school setting where students can participate as the “citizen scientists”. The findings suggest that further investigation of this citizen science approach in the elementary and middle school settings is warranted.

## Abbreviations

DT: Discovery Tool
SCCPHD: Santa Clara County Public Health Department
SRTS: Safe Routes to School
